# Evaluation of Metalloproteinase-8 Levels in Crevicular Fluid of Patients with Healthy Implants or Periodontitis

**DOI:** 10.1155/2017/4920847

**Published:** 2017-07-05

**Authors:** Paweł Aleksandrowicz, Paulina Żelechowska, Justyna Agier, Katarzyna Starska, Krzysztof Kędzierski, Joanna Wysokińska-Miszczuk, Ewa Brzezińska-Błaszczyk

**Affiliations:** ^1^Department of Periodontology, Medical University of Lublin, Lublin, Poland; ^2^Department of Experimental Immunology, Medical University of Lodz, Lodz, Poland; ^3^I Department of Otolaryngology and Laryngological Oncology, Medical University of Lodz, Lodz, Poland

## Abstract

Evaluation of periodontal and peri-implant tissue condition is mainly based on clinical examination and imaging diagnostics. Some data imply that Metalloproteinase-8 (MMP-8) level examination in peri-implant sulcular fluid (PISF) might be useful for evaluating the condition of peri-implant tissues and monitoring a development of peri-implant inflammation, including both mucositis and peri-implantitis. Hence, in this study, we decided to evaluate the level of MMP-8 in PISF obtained from patients without clinical symptoms of mucositis or peri-implantitis and compare it with MMP-8 level in gingival crevicular fluid (GCF) obtained from patients with healthy periodontium and those with varying severity of periodontitis. A total of 189 subjects were included in the study, and GCF/PISF samples were analysed for MMP-8 level by ELISA test. We documented that MMP-8 level in PISF obtained from patients without symptoms of mucositis or peri-implantitis was significantly higher not only than in GCF of periodontally healthy patients but also, which seems to be very interesting, than in GCF of patients with varying degrees of periodontal inflammation, consistent with earlier studies. Our observation might imply that monitoring of MMP-8 level in PISF could help to diagnose mucositis/peri-implantitis in an early stage, prior to clinical manifestations, which may allow for quick start of appropriate therapy.

## 1. Introduction

The dynamic development of modern implant dentistry has been present for more than 35 years. Various dental implant systems have been elaborated, and surgical and prosthetic techniques have been improved. Current data indicate that the percentage of successful implant treatment is very high [1, 2]. However, long-term studies have demonstrated that in some patients, the development of mucositis around implants, as well as peri-implantitis, may occur [3]. In extensive studies, Renvert et al. [4] documented that in 213 patients with 976 implants, peri-implant mucositis occurred in 59% and peri-implantitis appeared in 14.9% of cases. The development of peri-implantitis is a serious problem since it might lead even to the loss of the implant.

The causes of the peri-implantitis development and progression of inflammation are very different. Factors such as oral cavity health, proper hygiene, smoking, and stress are certainly important. Without a doubt, anatomical factors and proper attachment of connective tissue and epithelium to the implant surface affect the maintenance of the implant and the development of possible inflammatory processes [5, 6]. The implant-bone interaction depends on many factors, including properties of the material from which the implant is made [7]. Another important factor is the quality of the implant surface—its chemical, physical, and mechanical features [8]. It has been shown that the development of the surface of titanium implants increases the potential of biomechanical contact at the implant-bone connection and affects the rate of protein adsorption [9]. The roughness of implant surface also modulates the adhesion of osteoblasts, increases their enzymatic activity, and affects the amount and type of proteins synthesized by them [10]. Clinical studies conducted in recent years showed that the plain etched and sandblasted surface of the implants may sometimes cause the formation of peri-implantitis [11]. Therefore, studies on the implants with a rough surface at the top of the implant and the mechanically prepared surface around the head of the implant are being conducted.

A biological and morphological barriers formed around the implants in the process of osseointegration effectively prevents against bacterial penetration into tissues. The damage of the structure and function of these barriers allows bacterial penetration into the tissues. Direct interaction of bacteria and their products with periodontal tissues is another highly important factor, inducing the inflammatory process [12, 13]. However, current studies indicate that the development of inflammation in the periodontal tissues is mainly determined by immune response to pathogens, and tissue damage is caused by different humoral factors produced by defensive cells, that is, various proinflammatory cytokines, including interleukin- (IL-) 1*β*, IL-1Ra, IL-6, tumor necrosis factors (TNF) and chemokines, neutrophil lysosomal enzymes, reactive oxygen species (ROS), and eicosanoids (prostaglandins, leukotrienes) [14–18]. Metalloproteinases (MMPs), synthesized by activated cells of periodontal tissues, also have a significant impact on the course of immunoinflammatory processes in the periodontium. These enzymes are involved in the degradation of extracellular matrix (ECM) proteins such as laminin, collagens, proteoglycans, or fibronectin which lead to increased migration of inflammatory cells and destruction of the tissue structure. As a result, the action of MMPs can result in the destruction of ligament and bone resorption. Collagenase MMP-1, MMP-13, and, in particular, collagenase 2 (MMP-8) play a special role in inflammatory and destructive processes in periodontium since the substrates for these enzymes are collagen types I, II, III, and IV which are important proteins of periodontal attachment apparatus and the soft tissues around implants [19–21].

Evaluation of the condition of periodontal and peri-implant tissues is mainly based on clinical examination and imaging diagnostics. Nowadays, it is believed that the measurement of the concentration of humoral factors of inflammation in gingival crevicular fluid (GCF) and peri-implant sulcular fluid (PISF) may be very helpful in assessing the severity of the inflammatory process within periodontal tissues, particularly in the early periodontitis and/or peri-implantitis [22–24]. The information in this field, especially in the case of peri-implantitis, is not sufficient. Thus, the aim of this study was to analyse the MMP-8 levels, the key collagenase responsible for the destruction of periodontal tissues, in GCF from patients with varying severity of periodontitis and in PISF from patients with healthy implants and no signs of mucositis or peri-implantitis.

## 2. Materials and Methods

### 2.1. Study Population

A total of 189 subjects (85 males, 104 females; aged 20–71 years) were enrolled from the Department of Periodontology of the Medical University of Lublin. Prior to participation, the purpose and procedures were fully explained to all subjects, and written informed consent was obtained from each patient, the procedure being in line with the Declaration of Helsinki. Complete medical and dental histories were obtained from all subjects. None of the patients had any systemic disorders or had used antibiotics and/or anti-inflammatory drugs within the last 3 months. None of the subjects had received periodontal treatment within the last 6 months. Only nonsmokers were included in this study.

The patients were diagnosed according to clinical and radiographic criteria. Clinical parameters including gingival index (GI), probing pocket depth (PD), clinical attachment level (CAL), and bleeding on probing (BOP) were recorded. PD and CAL values were obtained using a conventional periodontal probe. All clinical data were recovered by one examiner. Based on the clinical data, the subjects were divided into five groups: (1) periodontally healthy subjects (13 males, 23 females) with no clinical evidence of gingival inflammation, no radiographic evidence of alveolar bone loss, and PD < 3 mm; (2) patients with mild periodontitis (18 males, 30 females) with PD 3-4 mm; (3) patients with moderate periodontitis (21 males, 22 females) with PD 4–6 mm; (4) patients with severe periodontitis (18 males, 12 females) with PD > 6 mm; and (5) periodontally healthy subjects (*N* = 32; 15 males, 17 females) who received implant treatment (implants with a new alternative hydrophilic surface SPI ELEMENT INICELL, Thommen Medical AG, Grenchen, Switzerland, and Brånemark System implant, Nobel Biocare, Gothenburg, Sweden). All subjects who had undergone a maxillary implant surgery were subjected to a laryngological examination in order to exclude paranasal sinus disorders and potential complications related to the above diseases. The bone density (D1, D2, D3, or D4) was established based on the clinical drilling resistance of the bone, according to Misch classification [25]. Implant examination of each patient generally included assessment of oral hygiene, PD, BOP, and mobility of implant. Survival of implants ranged from 36 to 147 months. Baseline characteristics of study groups are shown in Table 1.

All patients were followed up for at least one year at a frequency of 6–18 months. In order to make a periodical assessment of bone levels, radiographic images were taken from all patients by well-trained technicians using the parallel method during every follow-up visit. Bone level measurements (radiographic image analyses) were calculated (by two precalibrated experienced dentists in a blind manner) and determined by the distances between the alveolar bone crest and the respective tooth cusp.

### 2.2. GCF/PISF Sampling and Processing

Prior to GCF collection, the supragingival plaque was carefully removed. GCF samples were collected from the mesiobuccal site. The sites to be sampled were isolated with cotton rolls and gently air dried. GCF samples were collected with sterile PerioPaper strips (Oraflow Inc., Plainview, NY, USA) that were inserted into the gingival crevice until mild resistance was felt and left in place for 30 s. Mechanical irritation was avoided and strips visually contaminated with blood were discarded. After GCF collection, strips were placed in Eppendorf vials and immediately frozen at −80°C until use.

Clinical examinations of the group of patients with implants were performed after removal of the supraconstructions. Sampling of PISF was performed minimum 18 months following the surgery using sterile PerioPaper strips that were inserted into the gingival crevice until mild resistance was felt and left in place for 30 s. The paper points were then transferred into Eppendorf tubes and then immediately stored in a temperature of −80°C.

The GCF/PISF samples were analysed for MMP-8 by an ELISA test (Quantikine R&D Systems Inc., Minneapolis, MN, USA). For GCF/PISF extraction, strips were placed in tubes containing 500 *μ*L of phosphate-buffered saline (pH 7.2) and the tubes were shaken gently for 1 h at room temperature. The strips were removed and the fluids assayed by ELISA for MMP-8. All ELISA procedure was carried out according to the manufacturer's instruction. The ELISA plates were then assessed spectrophotometrically at an optical density of 450 nm. The MMP-8 determination was carried out in duplicate for each sample. The GCF/PISF MMP-8 concentrations were calculated from the standard curve. MMP-8 was determined as the total amount per sample.

### 2.3. Statistical Analysis

The statistical analysis for this study was performed using the package Statistica 12.5 (StatSoft Inc., USA). Spearman's rank correlation coefficient was carried out to analyse correlations between MMP-8 level and functioning period of implant as well as between MMP-8 level and bone quality. Normality of distribution was tested with the Shapiro-Wilk test. The levels of MMP-8 in GCF/PISF were compared using Mann–Whitney *U* test. A *P* value of <0.05 was considered statistically significant.

## 3. Results

**Figure 1:**
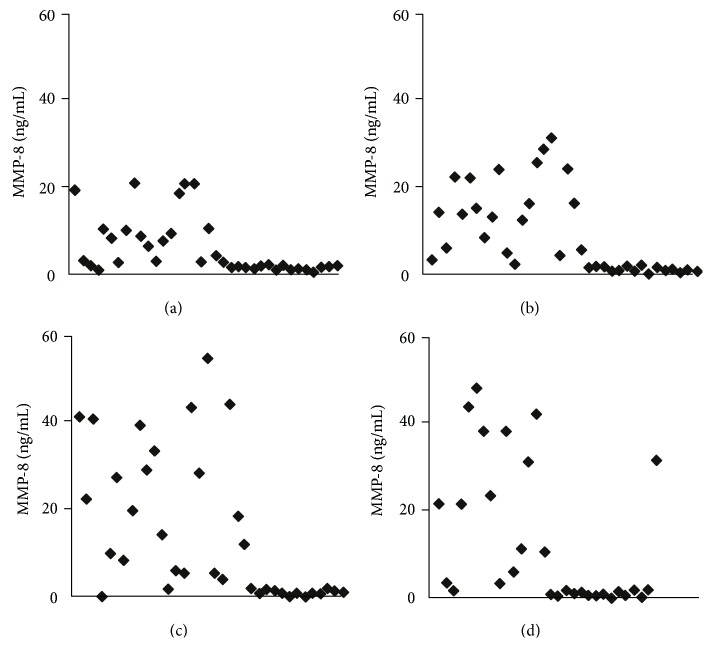


MMP-8 levels in GCF in patients with healthy periodontium and in patients with varying severity of periodontitis are shown in Figure 1. It was observed that the concentration of MMP-8 in each group of patients varied significantly, that is, from 0.21 to 20.70 ng/mL in patients with healthy periodontium, from 0.20 to 31.50 ng/mL in patients with mild periodontitis, from 0.34 to 54.90 ng/mL in patients with moderate periodontitis, and finally from 0.20 to 48.51 ng/mL in patients with severe periodontitis.

The MMP-8 level in PISF was highly different and ranged from 0.3 to 347.0 ng/mL (Table 2). No correlation between MMP-8 level in PISF and the duration of the implant functioning was observed. Besides, there was no connection between MMP-8 level in PISF and the type of prosthetic work on endosteal implants. Finally, no correlation between the MMP-8 level in PISF and bone density was noted, either.

The mean level of MMP-8 (±SD) in PISF was high and reached 40.46 ± 80.28 ng/mL, whereas the mean MMP-8 level in GCF in subjects with healthy periodontium was 5.61 ± 6.55 ng/mL. In patients with mild periodontitis, moderate periodontitis, and severe periodontitis, the MMP-8 levels in GCF were 7.40 ± 9.07 ng/mL, 12.43 ± 10.06 ng/mL, and 13.17 ± 16.43 ng/mL, respectively. A statistical analysis revealed that MMP-8 level in PISF was significantly higher not only than in periodontally healthy subjects (*P* = 0.0099) but also than in patients with mild periodontitis (*P* = 0.0321), moderate periodontitis (*P* = 0.0489), and, which should be pointed out, with severe periodontitis (*P* = 0.0255). What is more, statistical analysis also revealed that MMP-8 levels in GCF in patients with mild, moderate, and severe periodontitis were statistically higher than those in periodontally healthy subjects (*P* = 0.0020, *P* = 0.0003, *P* = 0.020, resp.) (Table 3).

## 4. Discussion

Postimplantation biological processes occurring in bone structures are the result of both resorption and osteogenesis. The process of implant osseointegration goes in several stages, and its last phase is the interior reconstruction of the bone, called bone remodeling. Bone remodeling is an active and dynamic process that relies on the correct balance between bone resorption by osteoclasts and bone deposition by osteoblasts. Moreover, these two functions must be tightly coupled not only quantitatively but also in time and space [26, 27]. It should be stressed that this continuous process of bone remodeling ensures a long-term implant's functionality. Bone turnover processes are regulated by various humoral factors, including a significant role of MMP-8 [28].

It is a long time since it has been recognized that the level and activity of MMP-8 evaluated in GCF may be a convenient and objective marker of the health assessment of periodontal tissue and of the evaluation of severity of the soft and hard tissue destruction. Some authors have indicated that in GCF obtained from patients with advanced or aggressive periodontitis, MMP-8 levels are significantly higher than in patients with a healthy periodontium [29–33]. Moreover, it is pointed out that the assessment of the MMP-8 levels in GCF can be an excellent indicator of the effects of the advanced periodontitis treatment [31, 34–36]. In our previous studies, we have documented that scaling and root planing (SRP) in patients with chronic periodontitis resulted in a significant decrease in MMP-8 concentration in GCF [29]. Some data imply that MMP-8 level in PISF might be useful for evaluating the condition of peri-implant tissues and monitoring a development of peri-implant inflammation—mucositis or peri-implantitis [37–41]. However, there is only little information on this issue and the results are ambiguous. Hence, in this study, we decided to evaluate the levels of the MMP-8 in PISF obtained from patients without clinical symptoms of mucositis or peri-implantitis and compare it with the level of MMP-8 in GCF obtained from patients with healthy periodontium and those with varying degrees of periodontitis. The results of this study have indicated that the levels of MMP-8 in the GCF from patients with various severity of periodontitis were significantly higher than in patients with healthy periodontium. Moreover, we have also noticed that patients with severe periodontitis demonstrated the highest MMP-8 level in GCF. These observations are consistent with those obtained by other authors. Additionally, we have documented that MMP-8 level in PISF obtained from the patients without symptoms of mucositis or peri-implantitis was significantly higher not only than in GCF of periodontally healthy patients but also, which seems to be very interesting, than in GCF of patients with varying severity of periodontitis.

The presence of MMP-8 in PISF obtained from patients with peri-implantitis or mucositis has been observed by many authors [37–40]. Janska et al. [41] have pointed out that the level of MMP-8 was lower in peri-implant mucositis than in progressive peri-implantitis, and the high level of this collagenase did not correlate with the severity of peri-implantitis. Ramseier et al. [42] have also found high levels of MMP-8 in PISF obtained from sites of mucosal inflammation. Kivelä-Rajamäki et al. [43] have observed increased concentrations of MMP-8 in PISF collected from peri-implantitis or mucositis sites. These observations seem to suggest that the assessment of the level/activity of MMP-8 in PISF might be useful in detection of developing peri-implant mucositis and peri-implantitis. What is more, Arakawa et al. [37] have further suggested that MMP-8 can be a good marker in evaluating progression of bone loss in peri-implatitis. Interestingly, MMP-8 was also detected in PISF of patients not affected by mucositis or peri-implantitis, although the level of this marker was always lower than in PISF collected from mucositis/peri-implantitis patients [38, 39, 43, 44]. Only Arakawa et al. [37] have not found the presence of MMP-8 in PISF collected from patients without clinical symptoms of inflammation around the implants, and Xu et al. [44] have found that the concentration of MMP-8 in PISF in healthy implants is very low. In our study, we have proved the presence of MMP-8 in PISF obtained from individuals without clinical symptoms of developing mucositis or peri-implantitis. What is more, we have also established that the concentration of this collagenase was significantly higher than its level in GCF obtained from patients with periodontitis, even with severe periodontitis.

## 5. Conclusion

It is well established that the initial stage of mucositis or peri-implantitis is asymptomatic. Thus, our observation might imply that monitoring of MMP-8 level in PISF could help to diagnose mucositis/peri-implantitis in an early stage, prior to clinical manifestations, which may allow for quick start of appropriate therapy.

## Figures and Tables

**Table 1:** Demographic and clinical characteristics of study population.

	Control	Mild periodontitis	Moderate periodontitis	Severe periodontitis	Implant
	(I)	(II)	(III)	(IV)	(V)
*N*	36	48	43	30	32
Age (years)					
Mean ± SD	35 ± 8	38 ± 9	40 ± 9	42 ± 10	52 ± 16
Range	20–51	27–58	27–60	28–59	20–71
Gender					
Male	13	18	21	18	15
Female	23	30	22	12	17
Total natural teeth					
Mean ± SD	29 ± 3	28 ± 3	27 ± 3	26 ± 3	14 ± 11
Range	24–32	20–32	22–32	22–32	0–29
Total implants					
Mean ± SD	0	0	0	0	6 ± 3
Range	—	—	—	—	1–12
PD (mm)					
Mean ± SD	1.64 ± 0.61	3.34 ± 0.39	4.49 ± 0.23	5.55 ± 0.42	2.84 ± 0.57
Range	0.2–2.5	3-4	4-5	4–6	1.9–4
CAL (mm)					
Mean ± SD	0.71 ± 1.20	1.14 ± 1.32	1.80 ± 1.60	6.48 ± 1.65	—
Range	0–4.5	0–4.5	0–6	1.5–10	
GI (mm)					
Mean ± SD	0.81 ± 0.98	1.27 ± 0.74	1.30 ± 0.6	1.67 ± 0.71	0.34 ± 0.55
Range	0–3	0–2	0–2	0–3	0–2

SD: standard deviation.

**Table 2:** MMP-8 levels in PISF of individual patients with implants.

Patient	MMP-8 level (ng/mL)	Type of restoration	Implant site	Functioning period (months)	Gender	Bone density
1	1.0	Single tooth implant	R-Max-1	56	M	D3
2	67.3	Bridge	L-Max-4	55	F	D2
3	20.3	Bridge	R-Max-3	55	F	D3
4	22.0	Bridge	R-Man-3	83	M	D1
5	0.9	Single tooth implant	L-Max-3	102	F	D4
6	347.0	Single tooth implant	R-Max-1	58	M	D3
7	1.5	Bridge	R-Man-3	84	F	D2
8	206.0	Bridge	L-Max-3	48	F	D3
9	25.4	Bridge	R-Max-4	42	M	D2
10	1.8	Single tooth implant	L-Max-4	47	M	D3
11	33.0	Bridge	L-Max-5	45	F	D3
12	271.0	Bridge	R-Max-5	74	M	D3
13	0.3	Bridge	L-Max-4	49	F	D3
14	8.1	Bridge	R-Man-2	49	F	D1
15	13.7	Bridge	L-Max-3	38	F	D3
16	0.5	Bridge	L-Man-4	147	F	D4
17	14.0	Bridge	R-Man-4	39	F	D1
18	46.0	Bridge	L-Max-3	48	F	D3
19	29.5	Bridge	R-Man-4	48	F	D2
20	2.1	Single tooth implant	R-Max-4	83	F	D3
21	5.4	Single tooth implant	L-Max-4	59	F	D3
22	2.1	Single tooth implant	R-Max-4	36	M	D3
23	11.1	Bridge	R-Max-5	79	M	D3
24	20.4	Single tooth implant	R-Max-4	46	M	D4
25	2.6	Single tooth implant	L-Man-4	46	M	D2
26	38.7	Single tooth implant	L-Max-4	45	M	D4
27	49.0	Single tooth implant	R-Max-4	45	M	D4
28	23.4	Single tooth implant	R-Man-6	43	F	D2
29	2.8	Single tooth implant	L-Man-6	79	M	D2
30	14.1	Bridge	L-Max-3	114	F	D4
31	6.9	Bridge	R-Man-3	114	F	D1
32	4.5	Single tooth implant	R-Man-6	59	F	D2

R: right; L: left; Max: maxilla; Man: mandible; 1: incisal; 2: incisal; 3: canine; 4: first premolar; 5: second premolar; 6: first molar.

**Table 3:** The comparison of MMP-8 levels in different patients' groups.

	Control	Mild periodontitis	Moderate periodontitis	Severe periodontitis	Implant
	(I)	(II)	(III)	(IV)	(V)
MMP-8 (ng/mL)					
Range	0.21–20.70	0.20–31.50	0.34–54.90	0.20–48.51	0.30–347.00
Mean ± SD	5.61 ± 6.55	7.40 ± 9.07	12.43 ± 16.01	13.17 ± 16.43	40.46 ± 80.29
I versus II, *P* = 0.0020 I versus III, *P* = 0.0003 I versus IV, *P* = 0.020 I versus V, *P* = 0.0099		II versus III, *P* = 0.0313 II versus IV, *P* = 0.13 II versus V, *P* = 0.0321		III versus IV, *P* = 0.357 III versus V, *P* = 0.0489 IV versus V, *P* = 0.0255	

SD: standard deviation.
